# Elevated plasma tyrosine kinases VEGF-D and HER4 in heart failure patients decrease after heart transplantation in association with improved haemodynamics

**DOI:** 10.1007/s00380-019-01548-1

**Published:** 2020-01-20

**Authors:** Salaheldin Ahmed, Abdulla Ahmed, Joanna Säleby, Habib Bouzina, Jakob Lundgren, Göran Rådegran

**Affiliations:** 1 Department of Clinical Sciences Lund, Cardiology, Lund University, Lund, Sweden; 2 The Haemodynamic Lab, The Section for Heart Failure and Valvular Disease, VO. Heart and Lung Medicine, Skåne University Hospital, Getingevägen 4, EA15, 22185 Lund, Sweden

**Keywords:** Haemodynamics, Heart failure, Heart transplantation, Pulmonary hypertension, Receptor protein-tyrosine kinases

## Abstract

Receptor tyrosine kinases (RTKs) are implicated in cardiovascular growth and remodelling. We aimed to identify the plasma levels of RTKs and related proteins and their association with haemodynamic alterations in heart failure (HF) and related pulmonary hypertension (PH) following heart transplantation (HT). Using proximity extension assay, 28 RTKs and related proteins were analysed in plasma from 20 healthy controls and 26 HF patients before and 1-year after HT. In end-stage HF, out of 28 RTKs, plasma vascular endothelial growth factor-D (VEGF-D) and human epidermal growth factor-4 (HER4) were elevated compared to controls (*p* < 0.001), but decreased (*p* < 0.0001) and normalised after HT. Following HT, plasma changes (Δ) of VEGF-D correlated with Δmean pulmonary artery pressure (*r*_s_ = 0.65, *p* = 0.00049), Δpulmonary artery wedge pressure (*r*_s_ = 0.72, *p* < 0.0001), Δpulmonary arterial compliance (PAC) (*r*_s_ = − 0.52, *p* = 0.0083) and Δpulmonary vascular resistance (PVR) (*r*_s_ = 0.58, *p* = 0.0032). ΔHER4 correlated with Δmean right atrial pressure (*r*_s_ = 0.51, *p* = 0.012), ΔNT-proBNP (*r*_s_ = 0.48, *p* = 0.016) and Δcardiac index (*r*_s_ = − 0.56, *p* = 0.0044). In HF patients following HT, normalisation of VEGF-D reflected reversal of passive pulmonary congestion and restored PAC and PVR; whereas the normalisation of HER4 reflected decreased volume overload and improved cardiac function. The precise function of these proteins, their potential clinical use and pathophysiological relation in HF and related PH remain to be elucidated.

**Electronic supplementary material:**

The online version of this article (10.1007/s00380-019-01548-1) contains supplementary material, which is available to authorized users.

## Introduction

Heart failure (HF), a global pandemic present in 1–2% of the adult population, is caused by pathological changes in structural and/or functional properties of the cardiomyocytes, cardiac interstitium, or both [1, 2]. Apart from pure haemodynamic overload, the progression of HF involves abnormal biochemical signalling of neurohormones, inflammatory cytokines and receptor tyrosine kinases (RTK), culminating into aberrant cardiac vascularisation and maladaptive left ventricular remodelling [2–4].

A frequent complication in HF irrespective of ejection fraction is pulmonary hypertension (PH) impacting negatively on symptoms and outcome. PH due to left heart disease (LHD) is mainly induced by left HF, congenital or valvular heart diseases and if sustained may lead to endothelial dysfunction, followed by excessive vasoconstriction and pulmonary vascular remodelling [5–7]. Moreover, such PH may advance into a “fixed” phenotype, harbouring stiff vessels unresponsive to pulmonary vasodilators, potentially imposing a relative contraindication for heart transplantation (HT) [7–9].

In the field of HF, blood borne biomarkers, including proteomics, are an area of immense interest and may in addition to their clinical utility, provide a profound pathophysiological understanding [8, 9]. Likewise, the most recent state-of-the-art review of PH pathology and pathobiology, highlight the importance of future biomarker research in the field of PH [10]. Noteworthy are RTKs and related proteins, involved in signalling pathways of vascular remodelling in PH, such as in pulmonary arterial hypertension (PAH), angiogenesis as well as in cardiac remodelling [10–12]. RTK are also emerging as potential prognostic and/or diagnostic targets in PH [10, 13, 14].

Although the principle aetiology of PH-LHD has been described, the precise underlying pathophysiology remains unclear and whether treatment of the pulmonary vascular component with PAH targeted therapies is beneficial, remains uncertain [15, 16]. Identifying new proteins related to the reversal of HF and associated PH with corresponding haemodynamic improvement may consequently aid in non-invasive and rapid detection of haemodynamic deterioration and in generating new hypotheses for experimental testing and clinical research. This may in turn offer new approaches for optimised treatments of HF and aid in future clinical decision making such as the incorporation of a multi-marker panel of biomarkers for personalisation of care [9, 17].

In search for new biomarkers potentially associated with the pathophysiology of HF and concomitant PH, we aimed to identify the plasma levels of RTKs and related proteins and their association with haemodynamic alterations in HF and related PH, before vs. after HT.

## Material and methods

### Study population

The present study, including those with PH-LHD, focused on end-stage left HF patients who were haemodynamically evaluated at Skåne University Hospital, Lund, Sweden between October 2011 and February 2016 and who approved to be included in a prospective cohort study and had blood samples collected before and during the 1-year follow-up after HT (*n* = 29). Amongst these, patients with missing postoperative haemodynamic data (*n* = 2) or with persistent PH after HT (*n* = 1) were excluded. Systolic and diastolic left ventricular dysfunction was diagnosed with echocardiography and/or magnetic resonance imaging and HF diagnosis was based on 2016 ESC guidelines [1]. The study also included 20 healthy controls, enrolled between December 2015 and March 2017, with no reported events of myocardial infarction, atrial fibrillation, stroke or diabetes mellitus. All participants were ≥ 18 years old. The study was conducted in accordance to the declaration of Helsinki and Istanbul. Ethical approval was obtained from the ethical board in Lund, Sweden (diary numbers: 2010/114; 2010/442; 2011/368; 2011/777; 2014/92 and 2015/270). Informed written consents were obtained individually from all participants.

### Protein analysis

Venous blood samples, collected between October 2011 and March 2017 from healthy controls and patients during clinical evaluations of HF during right heart catheterisation (RHC) before and 1-year after HT, were utilised. The samples were stored at −80 °C in the Lund Cardio Pulmonary Register (LCPR) cohort of Region Skåne’s biobank, initiated 2011. There was no time separation between blood sampling and the haemodynamic assessments before and 1-year after HT.

Plasma levels of NT-proBNP and 28 proteins belonging or related to RTK were analysed with proximity extension assay (PEA), which utilises pairs of protein specific oligonucleotide-linked antibodies and involves qPCR, to detect and quantify target proteins (Proseek Multiplex Cardiovascular II, III and Oncology II kits, Olink Proteomics, Uppsala, Sweden) [18]. All panels are validated regarding sensitivity, dynamic range, specificity, precision (repeatability and reproducibility) and scalability. More information can be found by downloading the panel specific validation data documents, www.olink.com/downloads. The proteins’ levels were expressed with linear NPX (Normalised Protein eXpression), a relative quantification scale in arbitrary units.

### Proteins

Plasma proteins were grouped according to receptors and associated ligands into (1) vascular endothelial growth factor (VEGF) family: platelet-derived growth factor subunit (PDGF)-A, PDGF-B, VEGF-A, VEGF-D, VEGF-receptor (VEGFR)-2, VEGFR-3, placenta growth factor (PlGF); (2) epidermal growth factor (EGF) family: amphiregulin, pro-epidermal growth factor (EGF), EGF receptor (EGFR), proheparin-binding EGF-like growth factor (HB-EGF), human epidermal growth factor receptor 2 (HER2), HER3, HER4, transforming growth factor alpha (TGF-α); (3) TEK family: angiopoietin-1, angiopoietin-1 receptor (Tie-2); (4) TAM receptor kinase (TAM) family: tyrosine-protein kinase Mer (MERTK), tyrosine-protein kinase receptor UFO (AXL); (5) ephrin receptor family: ephrin type-A receptor 2 (EphA2), ephrin type-B receptor 4 (EphB4); (6) intracellular tyrosine kinase family: tyrosine-protein kinase ABL1 (ABL1), tyrosine-protein kinase Lyn (LYN), proto-oncogene tyrosine-protein kinase Src (SRC); (7) other tyrosine kinases: fibroblast growth factor-binding protein 1 (FGF-BP1), hepatocyte growth factor (HGF), proto-oncogene tyrosine-protein kinase receptor Ret (RET) and stem cell factor (SCF).

### Haemodynamic assessment

Haemodynamics were evaluated in patients by RHC in supine position, by inserting a Swan Ganz catheter (Baxter Health Care Corp, Santa Ana, CA) through an introducer predominantly into the right internal jugular vein. Acquired parameters included mean-, systolic- and diastolic pulmonary artery pressures (mPAP, sPAP, dPAP), mean right atrial pressure (MRAP) and pulmonary artery wedge pressure (PAWP). Heart rate (HR) and cardiac output (CO) were obtained by electrocardiography and thermodilution, respectively. Mixed venous oxygen saturation (SvO_2_) was measured from the pulmonary artery and arterial oxygen saturation (SaO_2_) from the radial artery. Arteriovenous oxygen difference a-vO_2_diff = (SaO_2_ − SvO_2_) × plasma haemoglobin × 1.34. Mean arterial pressure (MAP) was measured noninvasively.

### Haemodynamic definitions and renal clearance

Calculated parameters include cardiac index (CI) = CO/body surface area (BSA), stroke volume (SV) = CO/HR, stroke volume index (SVI) = SV/BSA, transpulmonary pressure gradient (TPG) = mPAP − PAWP, pulmonary vascular resistance (PVR) = TPG/CO, PVR index (PVRI) = TPG/CI, diastolic pulmonary pressure gradient (DPG) = dPAP – PAWP, right ventricular stroke work index (RVSWI) = (mPAP − MRAP) × SVI, left ventricular stroke work index (LVSWI) = (MAP − PAWP) × SVI and pulmonary arterial compliance (PAC) = SV/(sPAP − dPAP). The creatinine-based estimation of glomerular filtration rate (eGFR) was calculated using the revised Lund-Malmö formula [19].

PH-LHD was diagnosed by cardiologists, defined according to guidelines as a resting mPAP ≥ 25 mmHg and a PAWP > 15 mmHg. This was further sub-classified into (1) isolated post-capillary PH (DPG < 7 mmHg and/or PVR ≤ 3 WU) or (2) combined post- and pre-capillary PH (DPG ≥ 7 and/or PVR > 3 WU) [5]. HT were performed in accordance with the International Society for Heart Lung Transplantation [20, 21]. The heart transplantation procedures were performed at Skåne University Hospital, Lund, Sweden.

### Patients’ characteristics

Patients’ characteristics before and 1-year after HT are shown in Table 1 as previously described [22]. Among the 26 included patients, three (11.5%) had diabetes mellitus, five (19%) had hypertension and 14 (54%) had a history of atrial fibrillation before HT. Haemodynamic characteristics of all patients (*n* = 26) and a subgroup (*n* = 19) with PH-LHD are described in Table 2 and Supplementary Table 1, respectively. MPAP, PAWP, MRAP, PVR, PVRI and a-vO_2_diff decreased, whereas MAP, CO, CI, SV, SVI, PAC, LVSWI and SvO_2_ increased after HT (*p* < 0.0003). Moreover, TPG, DPG, HR and RVSWI did not change before vs. 1-year after HT (FDR < 0.01). Patients’ eGFR did not differ before vs. after HT (*p* = 0.15). In addition, the 20 controls had a median (IQR) age of 41 (26.8–50.5) years, MAP 89 (95–100) mmHg, BSA 1.92 (1.75–1.99) m^2^ (*n* = 19) and 10 (50%) were female.

**Table 1:** Characteristics of patients before and 1-year after heart transplantation

Variable	Pre-HT (*n* = 26)		Post-HT (*n* = 26)	
	*n*	Median (IQR)	*n*	Median (IQR)
Female *n* (%)	5 (19.2)			
Age (years)	26	50 (45–61)^a^	26	52 (47–63)
Height (cm)	26	178 (172–180)	26	177 (172–181)
Weight (kg)	25	80 (71–89)	26	78 (69–90)
BSA (m^2^)	25	2 (1.8–2.1)	26	2 (1.8–2.1)
Creatinine (μmol/L)	25	108 (90–123)	26	114 (97–142)^b^
eGFR (mL/min/1.73 m^2^)	25	63 (55–71)	26	53 (43–72)^b^
Atrial fibrillation *n* (%)	26	13 (50)	26	–
Hypertension *n* (%)	26	5 (19.2)	26	3 (11.5)
Diabetes mellitus *n* (%)	26	1 (3.8)	26	9 (34.6)

	*n* (%)	*n* (%)
HF and PH classification		
HFrEF	24 (92.3)	–
HFpEF	2 (7.7)	–
PH	19 (73.1)^c^	–
IpC-PH	10 (38.5)	–
CpC-PH	9 (34.6)	–
HF aetiology		
DCM	17 (65.4)	–
HCM	3 (11.5)	–
ICM	3 (11.5)	–
Other	3 (11.5)	–
Medications		
β-blockers	25 (96.2)	9 (34.6)
ACEi	11 (42.3)	–
ARB	11 (42.3)	10 (38.5)
MRA	22 (84.6)	3 (11.5)
Furosemide	24 (92.3)	12 (46.2)
Cordarone	4 (15.4)	–
Prednisolone	1 (3.8)	25 (96.2)
Cyclosporine	–	3 (11.5)
Tacrolimus	–	23 (88.5)
Mycophenolate mofetil	–	21 (80.8)
Azathioprine	–	5 (19.2)

*HT* heart transplantation, *IQR* interquartile range, *BSA* body surface area = (weight^0.425^ × height^0.725^) × 0.007184 [55], *PH* pulmonary hypertension, *HFrEF and HFpEF* heart failure with reduced (EF < 50%) and preserved ejection fraction (EF ≥ 50%), *IpC-PH* isolated post-capillary PH, *CpC-PH* combined post and pre-capillary PH, *DCM* dilated cardiomyopathy, *HCM* hypertrophic CM, *ICM* ischemic CM, *ACEi* angiotensin converting enzyme inhibitor, *ARB* angiotensin receptor blocker, *MRA* mineralocorticoid receptor antagonist

^a^*p* < 0.0001, FDR < 0.01; vs. control

^b^Nonsignificant vs. Pre-HT

^c^One patient could not complete the right heart catheterisation due to severe orthopnea. After optimisation with furosemide and levosimendan, subsequent right heart catheterisation confirmed IpC-PH

**Table 2:** Patients’ haemodynamic data before and 1-year after heart transplantation

Haemodynamic parameter	Pre-HT (*n* = 26)		Post-HT (*n* = 26)		Δ (Post-HT)–(Pre-HT) (*n* = 26)		*p* value
	*n*	Median (IQR)	*n*	Median (IQR)	*n*	Median (IQR)	Post-HT vs. Pre-HT
MAP (mmHg)	25	82 (77–93)	26	102 (91 to 108)	25	15 (9 to 27)	1.2 × 10^−6^*
mPAP (mmHg)	25	29 (24–38)	26	14 (12 to 17)	25	− 15 (−26 to −7.5)	1.8 × 10^−7^*
PAWP (mmHg)	24	20 (18–25)	26	7 (4 to 9.3)	24	− 17 (− 21 to − 6.5)	2.4 × 10^−7^*
MRAP (mmHg)	25	14 (7.5–18)	25	3 (1 to 4)	24	− 12 (− 15 to − 3.3)	4.4 × 10^−6^*
TPG (mmHg)	24	8.5 (6–12)	26	8 (5 to 10)	24	− 1.5 (− 6 to 2)	0.17
DPG (mmHg)	24	1 (0–3.8)	26	2 (− 0.25 to 4)	24	0 (− 2 to 3.5)	0.8
HR (beats/min)	25	73 (69–82)	26	82 (73 to 89)	25	7 (− 4 to 15)	0.063
CO (L/min)	25	3.3 (2.6–4.1)	26	5.5 (5 to 6.5)	25	2.2 (1.2 to 2.9)	6 × 10^−8^*
CI (L/min/m^2^)	25	1.8 (1.4–2.2)	26	2.8 (2.6 to 3.2)	25	1.1 (0.65 to 1.6)	1.2 × 10^−7^*
SV (mL/beat)	25	48 (35–58)	26	72 (66 to 78)	25	23 (14 to 34)	4.2 × 10^−7^*
SVI (mL/beat/m^2^)	25	25 (18–29)	26	36 (33 to 40)	25	12 (6.5 to 18)	1.2 × 10^−7^*
PVR (WU)	24	2.4 (1.4–3.5)	26	1.4 (0.89 to 1.9)	24	− 1.3 (− 1.9 to − 0.036)	6.5 × 10^−5^*
PVR index (WU/m^2^)	24	5.1 (2.9–6.9)	26	2.8 (1.7 to 3.7)	24	− 2.4 (− 4 to − 0.42)	5.3 × 10^−5^*
PAC (mL/mmHg)	25	2.2 (1.8–3.1)	26	5.4 (4.1 to 6.6)	25	3.2 (1.3 to 4)	0.00029*
LVSWI (mmHg × mL/m^2^)	24	1541 (1052–2007)	26	3344 (3167 to 3810)	24	1675 (1224 to 2532)	1.2 × 10^−7^*
RVSWI (mmHg × mL/m^2^)	25	362 (294–615)	25	429 (317 to 516)	24	62 (− 119 to 245)	0.64
SaO_2_ (%)	25	96 (94–97)	23	97 (96 to 98)	22	1.7 (− 0.2 to 2.8)	0.046
SvO_2_ (%)	25	52 (47–60)	26	69 (66 to 72)	25	17 (11 to 24)	8.3 × 10^−7^*
a-vO_2_diff (mL O_2_/L)	25	74 (63–81)	23	42 (40 to 51)	22	− 32 (− 40 to − 19)	2.4 × 10^−6^*

A single CO value was calculated by indirect fick before HT. Δ indicate parameter development following HT

*IQR* interquartile range, *WU* wood unit, *MAP* mean artery pressure, *mPAP* mean pulmonary artery pressure, *PAWP* pulmonary artery wedge pressure, *MRAP* mean right atrial pressure, *TPG* transpulmonary pressure gradient, *DPG* diastolic pressure gradient, *HR* heart rate, *CO* cardiac output, *CI* cardiac index, *SV* stroke volume, *SVI* stroke volume index, *PVR* pulmonary vascular resistance, *PAC* pulmonary arterial compliance, *LVSWI* left ventricular stroke work index, *RVSWI* right ventricular stroke work index, *SaO*_*2*_ arterial oxygen saturation, *SvO*_*2*_ venous oxygen saturation, *a-vO*_*2*_*diff* arteriovenous oxygen difference

*p < 0.0003, FDR < 0.01

### Statistics

Given the small population and dominance of non-parametric data, Wilcoxon signed-rank test, Mann–Whitney *U* test and Spearman’s rank correlation were used as appropriate. Values are presented as median [interquartile range (IQR)], unless otherwise stated. The two-stage step-up procedure of false discovery rate (FDR) was used to adjust for mass significance [23]. Two-sided *p* values lower than FDR thresholds were considered statistically significant. Statistical analyses were performed using Prism version 8.01 for Windows, GraphPad Software, La Jolla California USA, www.graphpad.com).

### Study setup

**Fig. 1:**
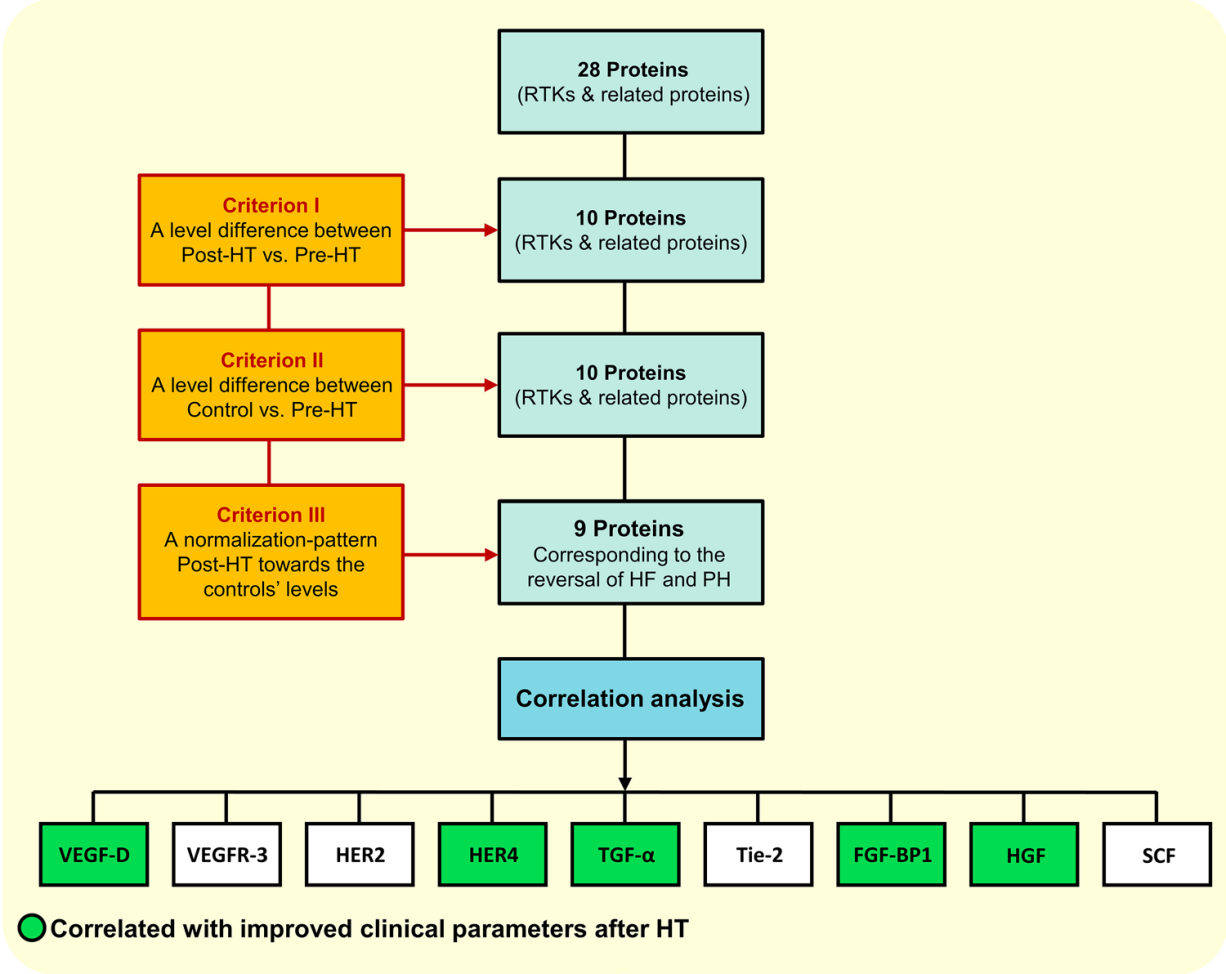
Selection of proteins corresponding to the reversal of heart failure and related pulmonary hypertension. *RTK* receptor tyrosine kinases, *HF* heart failure, *PH* pulmonary hypertension. Proteins’ abbreviations as in Table 2. False discovery rate (FDR) was used to accommodate for mass significance, (criterion I–III, FDR < 0.01); (correlation analysis, FDR < 0.1)

Three protein level specific comparisons were made, control vs. pre-HT, control vs. post-HT and post-HT vs. pre-HT. To select relevant proteins related to the reversal of HF and PH, three inclusion criteria were set: (1) a significant difference between post-HT vs. pre-HT, (2) a significant difference between control vs. pre-HT, as well as (3) a level change resembling a normalisation pattern towards controls’ levels after HT (FDR < 0.01). Proteins meeting all inclusion criteria were tested for correlation with NT-proBNP and haemodynamic parameters reflecting pulmonary passive congestion as well as right and left sided cardiac parameters, i.e. mPAP, PAC, PAWP, PVR, MRAP, CI and LVSWI (FDR < 0.1). Proteins meeting all three criteria and reflecting haemodynamic alterations were of specific interest. Next, specific correlations within the pre-HT group between proteins’ levels and haemodynamics were made. The study setup is summarised in (Fig. 1).

## Results

### Protein selection

The proteins’ baseline values and plasma alterations following HT are presented in Table 3. Out of 28 RTKs, nine proteins met all three inclusion criteria (Fig. 1), of which VEGF-D and HER4 were associated with the largest number of haemodynamic parameters.

**Table 3:** Proteins’ alterations in controls and patients before and 1-year after heart transplantation

Protein (AU)	Control (*n* = 20)		Pre-HT (*n* = 26)		Post-HT (*n* = 26)		Δ (Post-HT)–(Pre-HT) (*n* = 26)		*p* value		
	*n*	Median (IQR)	*n*	Median (IQR)	*n*	Median (IQR)	*n*	Median (IQR)	Pre-HT vs. C	Post-HT vs. C	Post vs. Pre-HT
NT-proBNP	20	1.1 (1.1–1.2)	26	24 (11–40)	26	2 (1.4–5.8)	26	− 17 (− 37 to − 8.4)	3.6 × 10^−13^*	5.0 × 10^−5^*	3.0 × 10^−8^*
**VEGF family**											
PDGF-A	20	7.4 (5.3–11)	26	7.4 (4.5–11)	26	5.2 (3.5–8.3)	26	− 1.4 (− 5.4 to 1.1)	0.82	0.055	0.071
PDGF-B	20	716 (404–965)	26	506 (228–802)	26	337 (220–707)	26	− 11 (− 289 to 239)	0.078	0.023	0.92
PlGF	20	120 (110–154)	26	188 (157–217)	26	203 (168–265)	26	14 (− 21 to 56)	2.6 × 10^−6^*	2.1 × 10^−9^*	0.32
VEGF-A	19	562 (490–692)	25	798 (666–1061)	26	847 (709–999)	25	− 14 (− 174 to 170)	4.7 × 10^−5^*	5.7 × 10^−6^*	0.65
VEGF-D	20	110 (94–121)	26	243 (199–262)	26	126 (92–163)	26	− 118 (− 153 to − 49)	5.0 × 10^−8^*	0.17	4.2 × 10^−7^*
VEGFR-2	19	96 (83–103)	25	72 (60–79)	26	73 (60–82)	25	− 0.3 (− 6.6 to 6)	3.7 × 10^−5^*	2.8 × 10^−5^*	0.99
VEGFR-3	19	53 (43–55)	25	59 (53–66)	26	44 (39–49)	25	− 16 (− 18 to − 9.2)	0.0051*	0.020	6.0 × 10^−8^*
**EGF family**											
Amphiregulin	19	2.7 (2.3–3)	25	5.4 (4–9.6)	26	4.1 (3.5–5.2)	25	− 1 (− 5 to 0.41)	1.3 × 10^−9^*	1.7 × 10^−7^*	0.020
EGFR	20	2.6 (2.4–2.9)	26	2.2 (2–2.4)	26	1.9 (1.8–2)	26	− 0.28 (− 0.45 to − 0.079)	0.0012*	0.011	0.045
HER2	19	87 (74–103)	25	106 (100–119)	26	79 (70–86)	25	− 30 (− 43 to − 20)	1.7 × 10^−5^*	6.8 × 10^−12^*	7.5 × 10^−6^*
HER3	19	177 (158–186)	25	172 (151–195)	26	170 (147–189)	25	− 6.2 (− 21 to 10)	0.64	0.385	0.84
HER4	19	23 (19–25)	25	29 (25–32)	26	20 (17–21)	25	− 9 (− 12 to − 5.7)	0.00021*	0.087	1.2 × 10^−7^*
EGF	19	395 (164–713)	25	132 (54–265)	26	163 (77–373)	25	42 (− 22 to 143)	0.80	0.67	0.30
HB-EGF	20	60 (43–81)	26	54 (41–77)	26	49 (43–74)	26	− 0.85 (− 17 to 14)	0.00014*	0.0088	1.2 × 10^−7^*
TGF-α	19	2.3 (1.9–2.6)	25	4.2 (3.7–4.9)	26	3.7 (2.9–4.2)	25	− 0.63 (− 1.6 to 0.24)	6.4 × 10^−9^*	6.7 × 10^−7^*	0.0051*
**TAM family**											
AXL	20	163 (145–198)	26	197 (164–249)	26	187 (166–261)	26	5.8 (− 47 to 29)	0.021	0.012	0.82
MERTK	20	12 (11–15)	26	18 (14–24)	26	16 (14–20)	26	− 1.2 (− 5 to 3.3)	3.1 × 10^−5^*	8.5 × 10^−5^*	0.15
**TEK family**											
Angiopoietin-1	20	306 (205–502)	26	295 (160–587)	26	213 (154–503)	26	− 38 (− 179 to 121)	0.80	0.31	0.58
Tie-2	20	185 (164–199)	26	273 (241–289)	26	184 (158–203)	26	− 87 (− 107 to − 67)	1.9 × 10^−8^*	0.97	6.0 × 10^−8^*
**Ephirns**											
EphA2	19	2.8 (2.6–3)	25	3.1 (2.8–4)	26	4.2 (3–5)	25	0.37 (− 0.03 to 1.3)	0.019	6.5 × 10^−5^*	0.019
EphB4	20	2.7 (2.4–2.9)	26	3 (2.5–3.7)	26	3.1 (2.5–3.7)	26	− 0.29 (− 0.65 to 0.2)	0.078	0.082	0.23
**Intracellular TK**											
ABL1	19	24 (8.3–40)	25	17 (11–29)	26	18 (13–28)	25	1.4 (− 9.4 to 8.5)	0.76	0.62	0.79
LYN	19	6.9 (4.4–9.3)	25	5 (3.3–6.5)	26	4.8 (3.1–6.9)	25	0.16 (− 2.2 to 1.6)	0.11	0.044	0.77
SRC	20	117 (109–138)	26	105 (52–147)	26	110 (68–162)	26	12 (− 18 to 46)	0.24	0.66	0.25
**Other TK**											
FGF-BP1	19	20 (18–20)	25	27 (19–36)	26	20 (17–22)	25	− 7.3 (− 14 to − 0.31)	0.0032*	0.97	0.00010*
HGF	19	45 (38–58)	25	110 (91–176)	26	58 (47–74)	25	− 59 (− 109 to − 29)	2.8 × 10^−10^*	0.014	1.2 × 10^−7^*
RET	19	16 (12–20)	25	14 (10–19)	26	12 (8.7–14)	25	− 1.4 (− 5.8 to − 0.23)	0.54	0.0094	0.0096
SCF	19	349 (324–446)	25	216 (161–263)	26	385 (343–428)	25	189 (86 to 237)	8.4 × 10^−6^*	0.53	6.0 × 10^−8^*

Plasma proteins were grouped according to receptors and associated ligands into: vascular endothelial growth factor (VEGF family); epidermal growth factor (EGF family); TAM receptor kinase (TAM-family); TIE angiopoietin receptor (TEK family); TK, tyrosine kinase. (Δ) Plasma level development following HT

*HT* heart transplantation, *C* control, *IQR* interquartile range. Proteins: *PDGF-A* platelet-derived growth factor subunit-A, *PDGF-B* platelet-derived growth factor subunit B, *PlGF* placenta growth factor, *VEGFR-2 and 3* VEGF receptor 2, 3, *EGF* pro-epidermal growth factor, *EGFR* epidermal growth factor receptor, *HB-EGF* proheparin-binding EGF-like growth factor, *HER2, 3 and 4* human epidermal growth factor receptor 2, 3, 4, *TGF-α* transforming growth factor alpha, *Tie-2* angiopoietin-1 receptor, *EphA2* ephrin type-A receptor 2, *EphB4* ephrin type-B receptor 4, *ABL1* tyrosine-protein kinase ABL1, *LYN* tyrosine-protein kinase Lyn, *SRC* proto-oncogene tyrosine-protein kinase Src, *FGF-BP1* fibroblast growth factor-binding protein 1, *HGF* hepatocyte growth factor, *RET* proto-oncogene tyrosine-protein kinase receptor Ret, *SCF* stem cell factor

*p < 0.01, FDR < 0.01

### Plasma VEGF-D and HER4 in end-stage heart failure patients

**Fig. 2:**
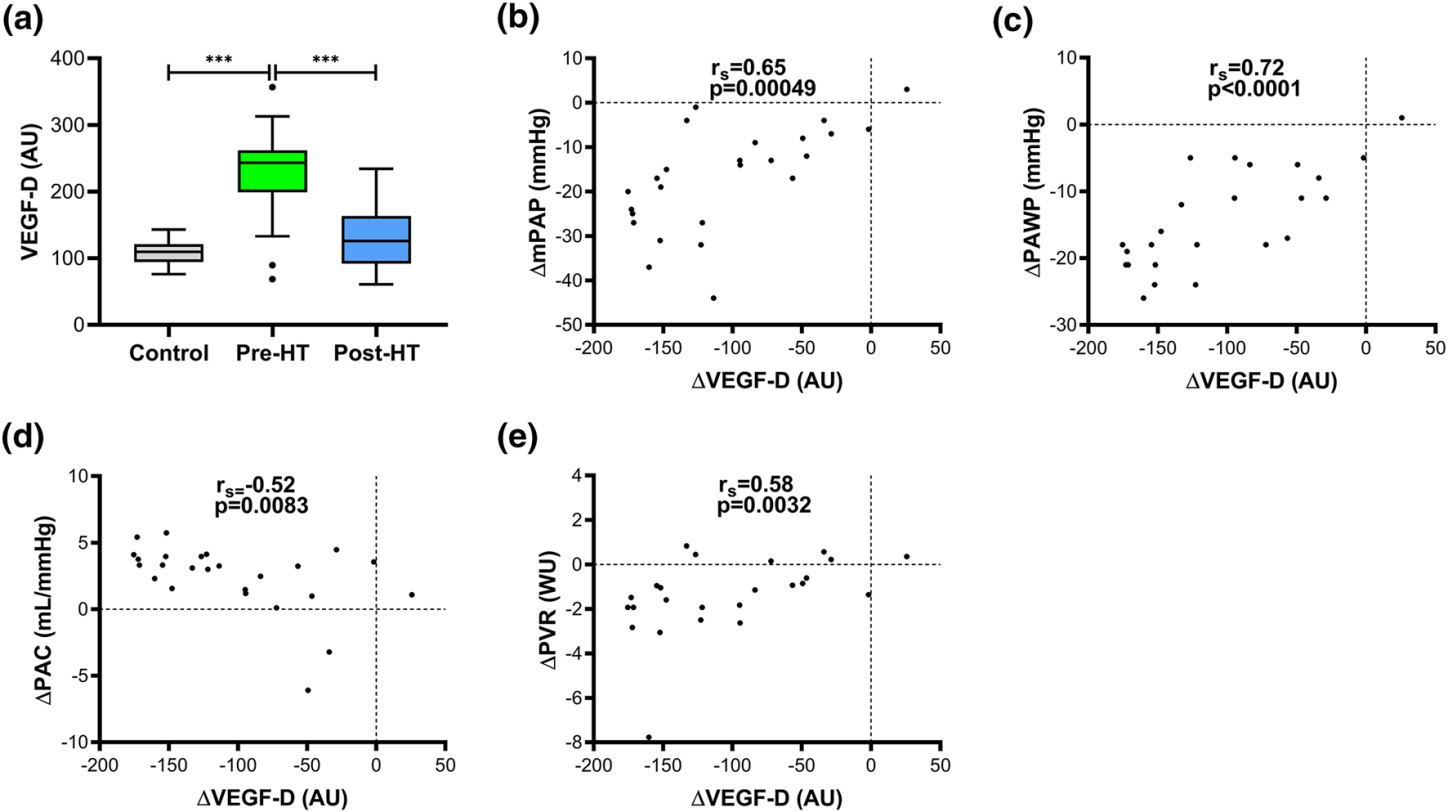
Plasma VEGF-D and correlations with haemodynamic changes following heart transplantation. Outliers calculated with Tukey’s fence. *HT* heart transplantation, *AU* arbitrary units, *r*_*s*_ Spearman’s correlation coefficient; ***p < 0.0001. Haemodynamic abbreviations as in Table 2. **a** VEGF-D levels in controls and heart failure patients before and 1-year after HT. The decrease (∆) of VEGF-D post-HT correlated with **b** ∆mPAP, **c** ∆PAWP, **d** ∆PAC and **e** PVR

**Fig. 3:**
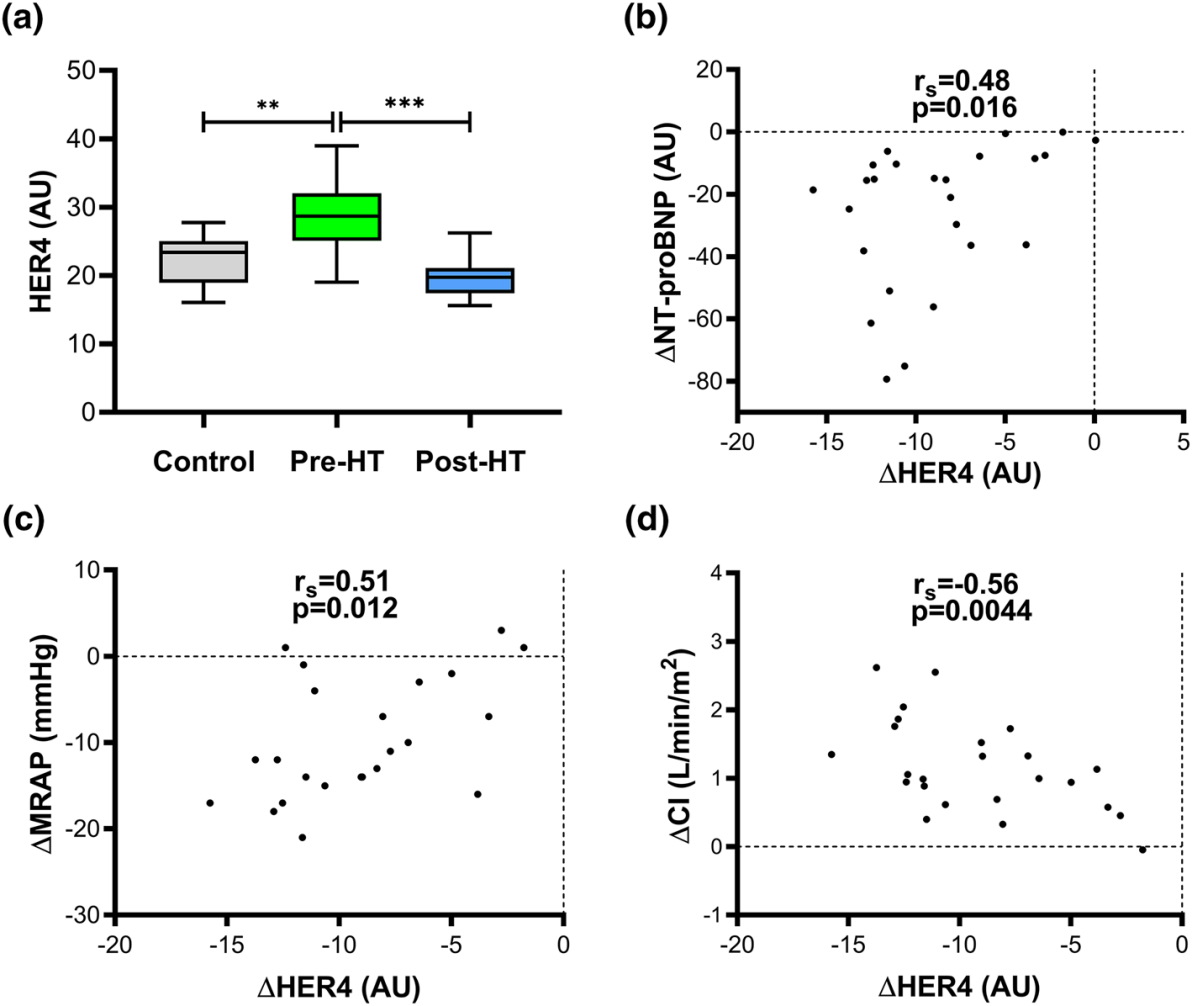
Plasma HER4 and correlations with haemodynamic changes following heart transplantation. Outliers calculated with Tukey’s fence. *HT* heart transplantation, *AU* arbitrary units, *r*_*s*_ Spearman’s correlation coefficient; **p < 0.001; ***p < 0.0001. Haemodynamic abbreviations as in Table 2. **a** HER4 levels in controls and heart failure patients before and 1-year after HT. The decrease (∆) of HER4 post-HT correlated with **b** ∆NT-proBNP, **c** ∆MRAP and **d** improved CI

In end-stage HF patients with or without PH, the plasma levels of VEGF-D (Fig. 2) and HER4 (Fig. 3) were elevated in comparison to controls (*p* < 0.001, FDR < 0.01). In response to HT, resolving pre-existing HF, these levels decreased (*p* < 0.0001, FDR < 0.01) and normalised, matching controls’ levels (Table 3).

### Plasma VEGF-D and HER4 level changes correlate with haemodynamic alterations

Spearman’s correlation coefficients and corresponding *p *values of associations between changes (∆) in proteins’ levels with ∆NT-proBNP and ∆haemodynamic parameters following HT are illustrated in Table 4. ∆VEGF-D (Fig. 2) correlated with ∆mPAP, ∆PAWP, ∆PAC and ∆PVR (*p* < 0.01; FDR < 0.1); whereas ∆HER4 (Fig. 3) correlated with ∆NT-proBNP, ∆MRAP and ∆CI (*p* < 0.02; FDR < 0.1). Baseline correlations of VEGF-D and HER4 with haemodynamic parameters before HT are described in Supplementary Table 2.

**Table 4:** Correlation analysis of changes (∆) between NT-proBNP and haemodynamics with proteins corresponding to the reversal of heart failure before and after heart transplantation

Δ Variables	mPAP (mmHg)		PAC (mL/mmHg)		PVR (WU)		PAWP (mmHg)		MRAP (mmHg)		NT-proBNP (AU)		CI (L/min/m^2^)		LVSWI (mmHg/mL/m^2^)	
	*n*	*r*_s_ (*p* value)	*n*	*r*_s_ (*p* value)	*n*	*r*_s_ (*p* value)	*n*	*r*_s_ (*p* value)	*n*	*r*_s_ (*p* value)	*n*	*r*_s_ (*p* value)	n	*r*_s_ (*p* value)	*n*	*r*_s_ (*p* value)
VEGF-D (AU)	25	0.65 (0.00049)*	25	− 0.52 (0.0083)*	24	0.58 (0.0032)*	24	0.72 (6.3 × 10^−5^)*	24	0.072 (0.74)	26	0.15 (0.48)	25	− 0.24 (0.24)	24	− 0.17 (0.42)
VEGFR-3 (AU)	24	0.32 (0.12)	24	− 0.25 (0.23)	23	0.42 (0.044)	23	0.15 (0.51)	23	− 0.028 (0.90)	25	0.12 (0.56)	24	− 0.27 (0.21)	23	0.029 (0.90)
HER2 (AU)	24	− 0.25 (0.25)	24	0.14 (0.52)	23	− 0.23 (0.30)	23	− 0.19 (0.39)	23	0.2 (0.36)	25	0.22 (0.29)	24	− 0.39 (0.060)	23	− 0.22 (0.31)
HER4 (AU)	24	0.18 (0.39)	24	0.22 (0.29)	23	0.22 (0.31)	23	0.0089 (0.97)	23	0.51 (0.012)*	25	0.48 (0.016)*	24	− 0.56 (0.0044)*	23	− 0.36 (0.096)
TGF-α (AU)	24	0.23 (0.29)	24	0.021 (0.92)	23	0.004 (0.99)	23	0.36 (0.095)	23	0.47 (0.022)	25	0.69 (0.00014)*	24	0.14 (0.51)	23	− 0.3 (0.17)
Tie-2 (AU)	25	0.029 (0.89)	25	0.036 (0.86)	24	0.037 (0.87)	24	− 0.13 (0.54)	24	0.16 (0.44)	26	0.21 (0.30)	25	− 0.41 (0.044)	24	− 0.0096 (0.96)
FGF-BP1 (AU)	24	0.4 (0.0555)	24	0.13 (0.54)	23	0.24 (0.28)	23	0.24 (0.27)	23	0.6 (0.0022)*	25	0.68 (0.0002)*	24	− 0.32 (0.13)	23	− 0.25 (0.25)
HGF (AU)	24	0.25 (0.24)	24	0.24 (0.26)	23	0.21 (0.34)	23	0.076 (0.73)	23	0.44 (0.037)	25	0.58 (0.0026)*	24	− 0.029 (0.89)	23	− 0.078 (0.72)
SCF (AU)	24	0.16 (0.46)	24	− 0.15 (0.48)	23	0.33 (0.13)	23	0.083 (0.71)	23	− 0.29 (0.18)	25	− 0.38 (0.063)	24	0.27 (0.20)	23	0.33 (0.12)

Proteins: *VEGF-D* vascular endothelial growth factor-D, *VEGFR-3* VEGF receptor-3, *HER2 and 4* human epidermal growth factor receptor 2, 4, *TGF-α* transforming growth factor alpha, *Tie-2* angiopoietin-1 receptor, *FGF-BP1* fibroblast growth factor-binding protein 1, *HGF* hepatocyte growth factor, *SCF* stem cell factor. Haemodynamics: *mPAP* mean pulmonary artery pressure, *PAC* pulmonary arterial compliance, *PVR* pulmonary vascular resistance, *PAWP* pulmonary artery wedge pressure, *MRAP* mean right atrial pressure, *CI* cardiac index, *LVSWI* left ventricular stroke work index, *n* number of pairs, *r*_*s*_ Spearman’s correlation coefficient

*p < 0.017, FDR < 0.1

### Other RTKs and related proteins in end-stage heart failure patients

The plasma levels of VEGFR-3, HER2, TGF-α, Tie-2, HGF and FGF-BP1 were elevated in patients with severe HF with or without PH, compared to healthy controls (*p* < 0.01, FDR < 0.01). In response to HT, resolving HF and PH, these levels decreased (*p* < 0.01, FDR < 0.01) towards controls’ levels. Plasma SCF were, however, low in HF patients in comparison to healthy controls (*p* < 0.0001, FDR < 0.01). These levels increased after HT (*p* < 0.01), matching the controls’ levels (Table 3). These seven proteins correlated either with few clinical parameters or none as described in Table 4. Baseline correlations between proteins and selected haemodynamic parameters before HT are described in Supplementary Table 2.

## Discussion

RTK signalling regulates diverse cellular functions through activation of the downstream mitogen-activated protein kinases, of which ERK1/2, ERK5 and JNK are pivotal in cardiac development, hypertrophy as well as in both physiological and pathophysiologic cardiac remodelling [3]. In the present study, among the 28 RTKs and related proteins, plasma VEGF-D and HER4 were elevated in severe HF patients compared to controls. The elevated plasma VEGF-D and HER4 normalised after HT, matching controls’ levels. The decrease of plasma VEGF-D after HT furthermore reflected reversed passive pulmonary congestion and possibly pulmonary vasoconstriction, with the latter indicated by correlations with PAC and PVR. Moreover, decreased HER4 after HT was associated with improved cardiac function and decreased volume overload. Identifying the precise functional roles of VEGF-D and HER4, as well as their clinical implications may be of great importance as these proteins may be utilised as potential biomarkers, with possible pathophysiological importance, in HF and related PH.

VEGF-D is known to be a potent mediator involved in lymph and regular angiogenesis, endothelial proliferation as well as vascular and cardiac remodelling, through VEGFR-3 and/or VEGFR-2 binding [24, 25]. It is expressed by a variety of tissues, most abundantly in heart, lungs and skeletal muscles [26]. VEGFR-3 is expressed in endothelial cells and crucial for primary vascular network maturation and early cardiovascular system development [27]. In a mouse model of chronic airway inflammation, elevated VEGF-D expression induced lymphatic sprouting and increased lymphatic drainage [28]. In the clinical field, plasma VEGF-D is furthermore a diagnostic and a severity marker of lymphangioleiomyomatosis [29].

Dyspnoea and pulmonary extravascular fluid accumulation are common, non-specific features in HF, particularly in PH-LHD due to backward transmission of elevated left sided filling pressures, increasing PAWP and mPAP [6, 30, 31]. In a clinical study of dyspnoeic patients, plasma VEGF-D correlated with NT-proBNP and elevated levels were most abundantly found in HF diagnosed patients. Accordingly, the authors hypothesised that the elevated plasma VEGF-D may be a marker of HF and pulmonary congestion, where VEGF-D upregulation may be involved in pulmonary vascular and/or cardiac remodelling [30]. Likewise, in a recent study, plasma VEGF-D correlated with PAWP and its elevation in HF patients was hypothesised to augment pulmonary lymphatic clearance and mitigate the symptoms of pulmonary congestion [32]. Interestingly, we found that plasma VEGFR-3 levels changed similarly to VEGF-D, which theoretically may reflect increased VEGF-D mediated signalling, thereby increased pulmonary lymphangiogenesis and lymphatic clearance, mitigating potential symptoms of pulmonary congestion. In the present study, we provide additional evidence that elevated plasma of VEGF-D levels in end-stage HF normalise along with the haemodynamics in response to HT. Furthermore, the decrease in plasma VEGF-D after HT correlated with ∆mPAP, ∆PAWP, ∆PVR and ∆PAC, indicating a decrease in passive pulmonary congestion and potentially, if existent, reversal of PAC and PVR towards a restored pulmonary vascular state. Therefore, VEGF-D may have the potential to be used as a biomarker to assess the haemodynamic strain and/or the severity of HF and PH-LHD as well as in the differentiation between isolated post and combined post and pre-capillary PH, as it correlated with mPAP, PAWP and PVR.

Furthermore, VEGF-mediated VEGFR-2 activation stimulates cardiomyocyte growth [33] and intramyocardially administrated VEGF-D facilitates therapeutic cardiac angiogenesis, increasing myocardial perfusion in patients with refractory angina [34]. Additionally, a meta-analysis showed that treatment with approved multiple tyrosine kinase inhibitors was associated with higher risk of congestive heart failure compared to non-tyrosine kinase treated [35]. In the present study, however, the plasma levels of VEGFR-2 did not change before vs. after HT and remained low compared to healthy controls. Thus, it remains uncertain whether VEGF-D mediated VEGFR-2 signalling impacts cardiac remodelling and/or facilitates favourable cardiac angiogenesis during HF.

**Fig. 4:**
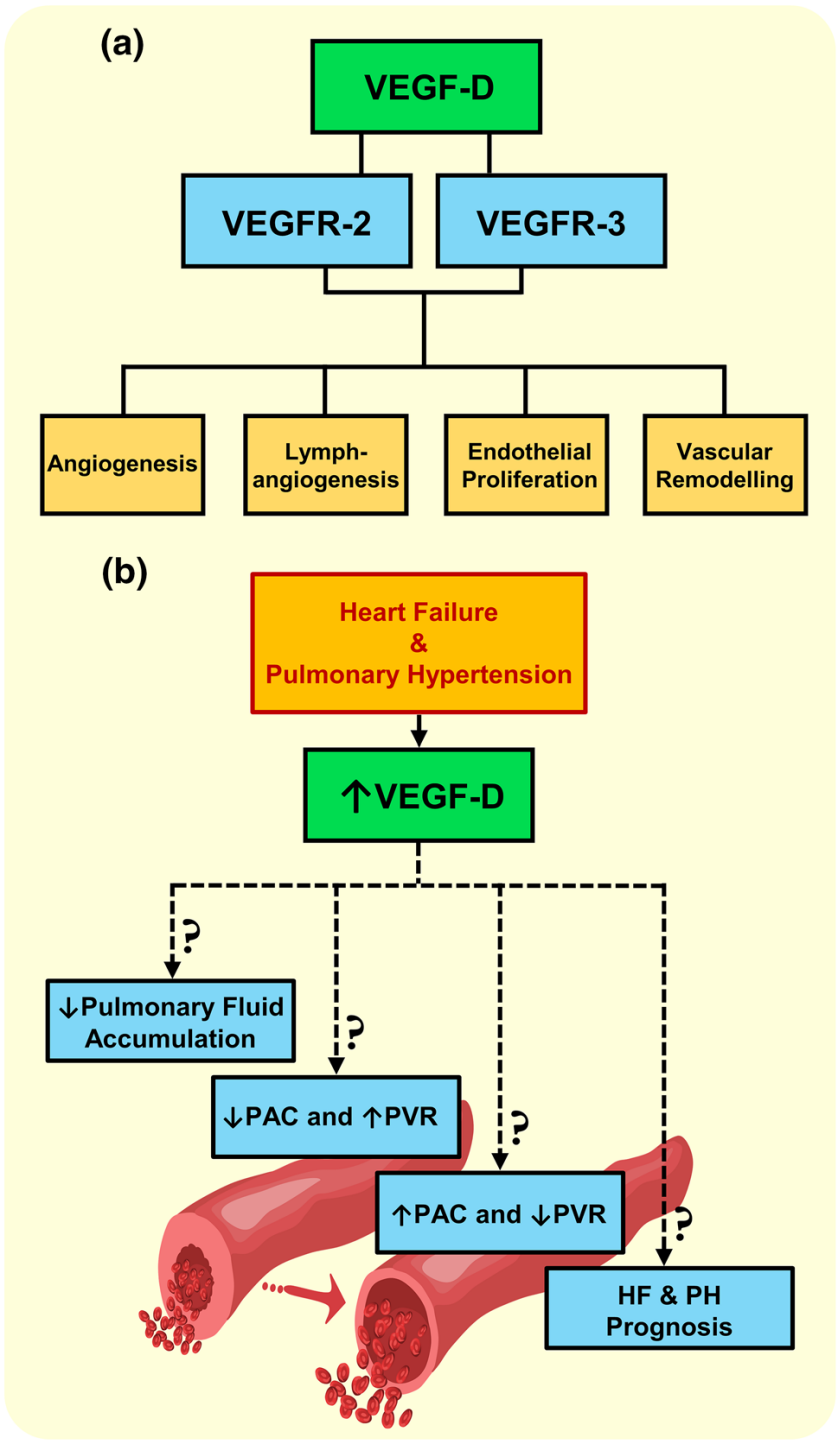
Illustration of vascular endothelial growth factor-D physiological roles and hypotheses. **a** Based on previous reports, the effects of VEGF-D mediated signalling through binding to its receptors. **b** Hypothetical roles and the possible clinical applicability of elevated plasma VEGF-D in heart failure (HF) and related pulmonary hypertension (PH). *PAC* pulmonary arterial compliance, *PVR* pulmonary vascular resistance

PH-LHD may ultimately lead to pulmonary vascular remodelling [6], which may be similar to, but not precisely the same as that observed in PAH patients [15, 36]. In the context of in vivo research, a model of severe angio-obliterative PAH displayed that expressions of VEGF-D and VEGFR-3 were elevated in lung tissue and inhibition of VEGFR-3 signalling prevented angio-obliteration, but did not reverse already existing vascular remodelling [37]. In clinical studies, high plasma VEGF-D differentiated PH-LHD from PAH, chronic thromboembolic PH (CTEPH) and controls, and a similar increase was related to PAH progression in systemic sclerosis patients [13, 14]. Further, we have recently confirmed in a larger patient cohort that apart from the levels being highest in HF patients with reduced ejection fraction with PH, plasma VEGF-D elevations were also found in PAH and CTEPH patients compared to healthy controls [38]. Hence, it is reasonable to hypothesise that VEGF-D and involved signalling pathways may have a role in the reversibility of PVR and PAC in HF patients with or without related PH. Noteworthy is, however, that the correlations between plasma VEGF-D with PAC and PVR may be explained by decongestion. The inverse hyperbolic PVR-PAC relationship is sensitive to acute and chronic PAWP elevations, which decrease PAC at a given PVR. This PAWP increase results in augmented total right ventricular pulsatile load, proposedly through enhancing pulmonary arterial pulse reflection, thereby increasing sPAP [39]. Collectively, whether VEGF-D elevation is a compensatory mechanism to ameliorate extravascular fluid accumulation or directly mediate a beneficial or pathological progression of PVR, PAC (independent of LV filling pressures) and/or vascular remodelling secondary to sustained pulmonary and cardiac congestion in HF and PH remain to be investigated. Moreover, VEGF-D may be of prognostic utility and aid in risk stratification of HF patients with passive pulmonary congestion, as PAC was proposed as a novel parameter in separating PH due to left HF patients at higher risk of death [40] and a strong predictor of mortality in HF with preserved ejection fraction [41] (Fig. 4).

HER4 signalling is pivotal in myocardial development, ventricular growth, proliferation and functional homeostasis of the adult heart and together with HER2 and their ligand Neuregulin-1 exert protective roles in the myocardium, e.g. enhancing cardiac repair mechanisms, reduce infarction-induced apoptosis and improve left ventricular ejection fraction [42]. Consequently, Neuregulin-1/HER2/HER4 signalling axis has recently emerged as a novel target to treat congestive HF and a current clinical trial is determining the efficacy of Neuregulin-1 administration (ClinicalTrials.gov, identifier NCT03388593). Herein, we demonstrate that plasma levels of HER2 and HER4 were high in severe HF compared to controls. These levels decreased post-HT, matching controls’ levels. Furthermore, the decrease in HER4 levels after HT correlated with ∆NT-proBNP, ∆MRAP and increased CI, reflecting reduced cardiac volume overload as well as improved cardiac function. In contrast to our results, another study showed that HER2 and HER4 tissue expressions are suppressed in the myocardium of HF patients in comparison to hearts from healthy controls [43]. This inconsistency can be explained by the fact that circulating receptors do not necessarily reflect a tissue-specific production, as the high plasma concentration in our study may originate from other tissues. Hypothetically, plasma elevation of HER2 and HER4 in response to HF and related haemodynamic worsening may slow HF progression by potentiating Neuregulin-1 effects. To note, however, receptors in plasma may operate differently than the membrane bound receptors and may exert antagonistic effects [44].

Other proteins, which either correlated with few clinical parameters or none, are FGF-BP1, TGF-α, HGF, SCF and Tie-2. These, in addition to the aforementioned proteins, may collectively be beneficial or related to the pathophysiological processes of HF. Noteworthy, SCF levels were low before HT, following an increase after HT towards controls’ levels. This may support that low plasma levels of SCF are associated with increased cardiovascular disease and mortality [45].

Limitations of our study include a small population and slightly younger controls. However, our study size corresponds to other HT studies, which has resulted in larger trials. A major strength is the use of PEA to assess plasma proteins’ levels. This method is highly sensitive, specific and avoids unspecific binding. Even if isoform specific detection of VEGF-D was absent, PEA is superior to traditional multiplex immunoassays [18]. False positive results may, however, be present due to the large number of statistical tests conducted despite adjusting for mass significance. Also, confounding factors such as medication intake, diurnal variations and other comorbidities may have influenced our results. The effects of postoperative withdrawal of HF medications (ACE-inhibitors, β-blockers and mineral corticoid receptor antagonists) on plasma proteins’ levels are unknown. However, it is established that ACE-inhibitors reduce ventricular wall stress and increase CO, whereas β-blockers enhance left ventricular geometry and function [46, 47]. Given that RTK signalling is involved in cardiac remodelling and hypertrophy [3], the use of ACE-inhibitors and β-blockers may thus have affected the proteins’ levels. Also, baseline VEGF-D levels correlated with mPAP, PAWP, PAC and PVR, which slightly allow to avoid the post-HT medication limitation. However, this is not apparent for HER4, as its baseline values only correlated with NT-proBNP and MRAP (Supplementary Table 2). Moreover, the use of angiotensin receptor blockers was almost equally distributed between the pre and post-HT groups, and therefore may not have affected our results. Although Everolimus and Sirolimus, (mTOR inhibitors) were not used in our cohort, these are known to inhibit angiogenesis, VEGF-D, VEGF-A and potentially VEGFR [48–51]. Tacrolimus inhibits VEGF, Angiopoietin-1 and Tie-2 [52]. Cyclosporine inhibits VEGF-mediated endothelial migration and angiogenesis [53]. Even though the role of steroids and cell cycle inhibitors remains unknown on plasma proteins’ levels, Mycophenolate mofetil has been proposed to downregulate downstream HER2 signalling pathways AKT, ERK and JAK-STAT3 [54]. Renal clearance, however, may only have had a minimal role in altering the proteins’ plasma concentrations in the transplant patients, as eGFR was unaltered following HT. Moreover, the present correlations between proteins and haemodynamics do not necessarily imply a causal relationship and should thus be interpreted with caution. Nevertheless, the present study is hypothesis generating and our findings may be of interest in developing a multi-marker panel for optimal future management of HF and related PH.

In conclusion, in HF patients, the decrease and normalisation of the elevated plasma VEGF-D after HT correlated with ∆mPAP, ∆PAWP, ∆PVR and ∆PAC, indicating VEGF-D to be a potential biomarker of reversed pulmonary passive congestion and possibly restored pulmonary vascular milieu. Moreover, the decrease of elevated plasma HER4 in HF patients correlated with ∆NT-proBNP, ∆MRAP and ∆CI, reflecting reduced cardiac volume overload and improved cardiac function in response to HT. Altogether, we encourage further research to elaborate the precise roles of these proteins and their clinical implications in HF and related PH as potential biomarkers of haemodynamic strain and/or HF/PH-LHD severity, for optimal clinical management of HF patients as well as better understanding of processes underlying the pathophysiology of HF.

## Electronic supplementary material

Below is the link to the electronic supplementary material.

Supplementary file1 (DOCX 25 kb)
