# Supplementary material for: Elevated plasma tyrosine kinases VEGF-D and HER4 in heart failure patients decrease after heart transplantation in association with improved haemodynamics
Source: Heart Vessels. 2020 Jan 20;35(6):786–99. doi: 10.1007/s00380-019-01548-1 (PMC7198637; doi:10.1007/s00380-019-01548-1)
Supplement: Supplementary file 1 — Supplementary file1 (DOCX 25 kb) [file 380_2019_1548_MOESM1_ESM.docx]

**Elevated Plasma Tyrosine Kinases VEGF-D and HER4 in Heart Failure Patients Decrease After Heart Transplantation in Association with Improved Hemodynamics**

**Journal name: Heart and Vessels**

**Authors:** Salaheldin Ahmed PhD student ^a,b^, Abdulla Ahmed PhD student ^a,b^, Joanna Säleby MD, PhD student ^a,b^, Habib Bouzina, PhD student ^a,b^, Jakob Lundgren MD, PhD ^a,b^ and Göran Rådegran, Associate Prof, DMSc, MD, MS Eng Phys ^a,b^

**Affiliations:** ^a^ Department of Clinical Sciences Lund, Cardiology, Lund University. ^b^ The Hemodynamic Lab, The section for Heart Failure and Valvular Disease, VO. Heart and Lung Medicine, Skåne University Hospital, Lund, Sweden.

**Corresponding author:** Salaheldin Ahmed

Address: Getingevägen 4, EA15, Skåne University Hospital, 22185 Lund, Sweden

E-Mail: [salaheldin.ahmed@med.lu.se](mailto:salaheldin.ahmed@med.lu.se)

Fax number: 0046 - (0)46 – 307984

Telephone: +46 72 927 57 29

| **Hemodynamic parameter** | **Pre-HT (n=19)** | | **Post-HT (n=19)** | |
| --- | --- | --- | --- | --- |
|  | n | Median (IQR) | n | Median (IQR) |
| MAP (mmHg) | 19 | 82 (78 ‒ 90) | 19 | 101 (90 ‒ 106) |
| mPAP (mmHg) | 19 | 31 (29 ‒ 39) | 19 | 13 (12 ‒ 17) |
| PAWP (mmHg) | 18 | 23 (19 ‒ 27) | 19 | 6 (4 ‒ 8) |
| MRAP (mmHg) | 19 | 14 (9 ‒ 17) | 18 | 2.5 (0 ‒ 4) |
| TPG (mmHg) | 18 | 10 (7 ‒ 14) | 19 | 8 (5 ‒ 10) |
| DPG (mmHg) | 18 | 2 (0 ‒ 4.3) | 19 | 2 (-1 ‒ 4) |
| HR (beats/min) | 19 | 73 (69 ‒ 78) | 19 | 81 (73 ‒ 87) |
| CO (L/min) | 19 | 3.2 (2.6 ‒ 4) | 19 | 5.4 (4.9 ‒ 6.5) |
| CI (L/min/m^2^) | 19 | 1.6 (1.4 ‒ 2.1) | 19 | 2.9 (2.6 ‒ 3.2) |
| SV (mL/beat) | 19 | 45 (34 ‒ 59) | 19 | 72 (66 ‒ 78) |
| SVI (mL/beat/m^2^) | 19 | 23 (18 ‒ 29) | 19 | 36 (34 ‒ 39) |
| PVR (WU) | 18 | 3.2 (2.3 ‒ 3.6) | 19 | 1.4 (0.9 ‒ 1.9) |
| PVR index (WU/m^2^) | 18 | 6.3 (4.7 ‒ 7.2) | 19 | 2.7 (1.8 ‒ 3.7) |
| PAC (mL/mmHg) | 19 | 1.8 (1.7 ‒ 3.1) | 19 | 5.1 (4 ‒ 6.3) |
| LVSWI (mmHg×mL/m^2^) | 17 | 1470 (934 ‒ 1841) | 19 | 3275 (3116 ‒ 3873) |
| RVSWI (mmHg×mL/m^2^) | 18 | 483 (296 ‒ 732) | 18 | 411 (306 ‒ 520) |
| SaO_2_ (%) | 19 | 96 (93 ‒ 96) | 17 | 97 (96 ‒ 98) |
| SvO_2_ (%) | 19 | 49 (46 ‒ 57) | 19 | 70 (67 ‒ 72) |
| a-vO_2_ diff (mL O_2_/L) | 19 | 74 (69 ‒ 82) | 17 | 42 (40 ‒ 51) |

**Supplementary Table 1. Hemodynamic alterations in PH-LHD patients before and one year after heart transplantation**

**Abbreviations:** PH-LHD, pulmonary hypertension due to left heart disease; IQR, interquartile range; WU, wood unit; MAP, mean artery pressure; mPAP, mean pulmonary artery pressure; PAWP, pulmonary artery wedge pressure; MRAP, mean right atrial pressure; TPG, transpulmonary pressure gradient; DPG, diastolic pressure gradient; HR, heart rate; CO, cardiac output; CI, cardiac index; SV, stroke volume; SVI, stroke volume index; PVR, pulmonary vascular resistance; PAC, pulmonary arterial compliance; LVSWI, left ventricular stroke work index; RVSWI, right ventricular stroke work index; SaO_2_, arterial oxygen saturation; SvO_2_, venous oxygen saturation; a-vO_2_ diff, arteriovenous oxygen difference.

**Supplementary Table 2. Baseline correlations between selected proteins, hemodynamic parameters and NT-proBNP**

| **Variable pre-HT** | **n (26)** | **r_s_ (p-value)** |
| --- | --- | --- |
| **VEGF-D** (AU) vs |  |  |
| mPAP (mmHg) | 25 | 0.54 (0.0052)* |
| PAWP (mmHg) | 24 | 0.42 (0.041)* |
| PAC (mL/mmHg) | 25 | -0.69 (0.00015)* |
| PVR (WU) | 24 | 0.66 (0.00048)* |
| **HER4** (AU) vs |  |  |
| MRAP (mmHg) | 24 | 0.35 (0.093)* |
| NT-proBNP (AU) | 25 | 0.35 (0.084)* |
| CI (L/min/m^2^) | 24 | -0.14 (0.51) |
| **TGF-α** (AU) vs |  |  |
| NT-proBNP (AU) | 25 | 0.66 (0.00029)* |
| **FGF-BP1** (AU) vs |  |  |
| MRAP (mmHg) | 24 | 0.68 (0.00028)* |
| NT-proBNP (AU) | 25 | 0.79 (2.7×10-6)* |
| **HGF** (AU) vs |  |  |
| NT-proBNP (AU) | 25 | 0.52 (0.0075)* |

(*), p-values considered significant; r_s_, spearman’s correlation coefficient; AU, arbitrary units and WU, wood units.

Abbreviations: **Proteins:** VEGF-D, vascular endothelial growth factor D; HER4, human epidermal growth factor receptor 4; TGF-α, transforming growth factor alpha; FGF-BP1 fibroblast growth factor-binding protein 1 and HGF, hepatocyte growth factor. **Hemodynamics:** mPAP, mean pulmonary artery pressure; PAC, pulmonary arterial compliance; PVR, pulmonary vascular resistance; PAWP, pulmonary artery wedge pressure, MRAP, mean right atrial pressure and CI cardiac index.
